# New chemolysis for urological calcium phosphate calculi – a study in vitro

**DOI:** 10.1186/1471-2490-5-9

**Published:** 2005-05-22

**Authors:** Zhang Xiang-bo, Wang Zhi-ping, Duan Jian-min, Lu Jian-zhong, Ma Bao-liang

**Affiliations:** 1 School of Life Sciences, LanZhou University, LanZhou, 730030, China; 2 Institute of Urology, the 2nd Hospital of LanZhou University, Cuiyingmen 80, LanZhou, 730030, China

## Abstract

**Background:**

Advances in techniques have left very few indications for open surgical extraction of urinary stones currently. These advances notwithstanding, the search continues for medical approaches to urinary stone management. In this study, we perform an in vitro study analyzing the efficiency and prospect of two new complex solutions in urological calcium phosphate calculi dissolution.

**Methods:**

Eighteen stones composed mainly of calcium phosphates were taken from patients who underwent kidney stone surgery. These stones were large enough (weight range 0.514–0.928 g) to be fragmented and matched equally into six groups. Chemolysis of phosphate stones was done with six different solvents and was repeated 3 times with 6 stones for each solution. At 24, 48 and 72 h, reduction in weight, percentage weight change, and dissolution rate; the dissolution rates at pH 5.0, 7.0 and 8.5 for each solution, using different cations (Na^+^, K^+ ^or Ca^2+^), according to different dilutions (1:1, 1:2, 1:3, 1:4) of S1 and S2 were simultaneously determined.

**Results:**

Calcium phosphate calculi were poorly dissolved by Phys and Art, and they had a low dissolution rate in pH 8.5 EDTA. The most effective solutions were S1, S2 and R, with 72 h mean dissolution rates: 5.75 ± 0.44 mg/hr (S1), 5.2 ± 0.63 mg/hr (S2), 4.55 ± 0.46 mg/hr (R) ( ± *s*, *p *< 0.01 R, S1 and S2 vs Phys, Art and EDTA; *p *< 0.05, S1 vs R, LSD-test). The mean percentage weight loss at 72 h was: 52.1 ± 15.75 % (S1), 44.4 ± 7.37 % (S2) and 40.5 ± 3.67 % (R) ( ± *s*, *p *< 0.01 R, S1 and S2 vs Phys, Art and EDTA, LSD-test). Diluted twice, S1 and S2 had even better effectiveness than their initial solution. The additive of Na^+^, K^+ ^or Ca^2+ ^greatly reduced the dissolution rates of S1, S2.

**Conclusion:**

Our data indicate that test solutions S1 and S2 are effective solvents in the chemolysis of calcium phosphate stones. At twice dilutions, these solutions are even more useful in the treatment of stone disease.

## Background

The most important phosphate-containing calculi involved in urinary stone disease are carbonate apatite, brushite, and struvite. Overall, phosphate stones account for 12–20% of all urinary stones and rank first in the list of recurrent calculi [1]. The most definitive therapy involving any type of phosphate stone is calculus removal by shock-wave lithotripsy, percutaneous stone removal, or open surgery (especially in children). However, while technologies have successfully treated patients with phosphate calculi, they are associated with injury to the urinary tissue and retained stone fragments have resulted in recurrences.

Stone dissolution through chemolysis remains an option for urologists even in the advent of more sophisticated modalities in the treatment of urolithiasis. Chemolysis via acidification of the urine and antibiotic therapy (especially for infection stones) are important adjuvant modalities for phosphate calculi therapy. Hitherto, a many solutions have been tested in the quest for more effective dissolution agents. The most effective solution among them is Renacidin (R), which is a buffer consisting mainly of citrate and gluconate [2-4]. These solutions are delivered directly into the kidney by ureteric catheter or percutaneous nephrostomy, and are successful in the dissolution of struvite. But the injury to urothelium restrains their wide use. So, solutions with more dissolution efficiency and less side effects are needed urgently and may be used widely. Our in vitro study was designed to evaluate the effectiveness of two new complex solutions (using D-gluconic acid-lactone, D-gluconic-acid and other ingredients which makes them different from R) in the chemolysis of calcium phosphate stones.

## Material and methods

### Stones specimen preparation

Eighteen stones composed mainly of calcium phosphate were taken from 18 patients who underwent kidney stone surgery. These stones were large enough (weight range from 0.514–0.928 g) to be fragmented and matched equally into six groups. Each stone was analyzed using the Merckognost at Urinary Stone analysis Kit. The chemical composition of these stones was varied but there was a predominance of calcium phosphate in almost all the stones and calcium oxalate was less than 10% (Table 1, Table 2).

**Table 1:** Chemical analysis of 18 stones for stone dissolution. There was a predominance of calcium phosphate in almost all the stones and calcium oxalate was less than 10%.

Stone%	Ca	Oxalate	NH_4_	PO_4_	Mg	Uric Acid	Tri-calcium phosphate	Calcium oxalate	Struvite
1.	45	2	2	57				7	
2.	60			45		10		9	
3.	60	10	4	55					10
4.	45	15		45		5		4	
5.	35	5		35					
6.	80	11	3	65				6	
7.	50	10		60				6	
8.	40	2	7	25		5		7	
9.	5	5		45	15		20	8	
10.		15		20	15		40	6	
11.	45	5	3	45		10			
12.	35	20	2	35					
13.	50	20	5	25					
14.	40	10		35		15			
15.	45	15		35				9	
16.	40	15	0.5	35		5			
17.	75	5		15					
18.	15	5		45		10			15

**Table 2:** Mean chemical composition of 18 stones, showing that there was a predominance of calcium phosphate in almost all the stones and calcium oxalate was less than 10%.

Composition	± *s *(%)
Ca^++^	42.5 ± 20.7
Oxalate	9.6 ± 6.2
NH_4_	1.5 ± 2.1
PO_4_	40.1 ± 13.9
Mg	1.7 ± 4.9
Uric acid	3.3 ± 4.9
Cystine	0.0 ± 0.0
Ca^++^oxalate	3.4 ± 3.7

### Preparation of solvent

Six different solvents were used for chemolysis of phosphate stones. The molecular formula and the molecular weights of some drugs used in these solvents are provided in Table 3.

**Table 3:** Molecular formulae and molecular weights of chemical drugs used in our experiment

Experimental drug	Molecular formula	Molecular weight
Citric acid	C_6_H_6_O_7_·H_2_O	210.14
Sodium citrate	Na_3 _C_6_H_6_O_7_·2H_2_O	294.11
D-Gluconic Acid Lactone	C_6_H_10_O_6_	178.1
Magnesium citrate	Mg_3_(C_6_H_5_O_7_)_2_·14H_2_O	703.4
Magnesium Carbonate	(MgCO_3_)_4_·Mg(OH)_2_·5H_2_O	485.8
Disodium EDTA	C_10_H_14_O_8_N_2_Na_2_·2H_2_O	372.24
Potassium citrate, tribasic	K_3_C_6_H_5_O_7_·H_2_O	324.34
Potassium phosphate, monobasic	KH_2_PO_4_	136.09

1. Physiologic sodium chloride solution (Phys).

2. Artificial urine (Art, pH5.7, according to Griffith et al [5]) consisting of urea (25 g/L), sodium chloride (4.6 g/L), potassium-dihydrogen-phosphate (2.8 g/L), sodium sulfate (2.3 g/L), potassium chloride (1.6 g/L), ammonium chloride (1.0 g/L), calcium chloride dehydrate (0.1 g/L), sodium oxalate (0.05 g/L), and sodium citrate (0.01 g/L), dissolved in 1000 ml. distilled water.

3. 0.03 M disodium ethylenediaminetetraacetic acid buffered to pH 8.5 with triethanolamine (EDTA, pH 8.5)

4. 10% Renacidin (R, pH 3.9), consisting of citric acid (28.2 g), gluconic acid (5.0 g), calcium carbonate (1.0 g), magnesium bicarbonate (14.5 g), citrate magnesium (2.5 g), dissolved in 1000 ml. distilled water.

5. Test solution 2 (S2, pH 4.0), prepared using citric acid (18.0 g), citrate magnesium (1.0 g), calcium carbonate (0.5 g), magnesium carbonate (7.5 g), D-gluconic acid-lactone (3.0 g), dissolved in 100 ml. distilled water. The solution was kept at 37? for 3 days, then diluted with 140 ml. distilled water. The concentrations of these ingredients are shown in Table 4.

**Table 4:** Concentrations of chemical drugs contained in S1 and S2

Chemical drug	Concentration (mmol/L)
Citric acid	357.1
Magnesium citrate	5.9
Magnesium Carbonate	64.3
D-Gluconic Acid Lactone	70.2
Calcium carbonate	20.8
D-Gluconic Acid	63.8
Sodium citrate	302.7
Potassium citrate, tribasic	302.7
Calcium chloride, anhydrous	454.5

Calcium carbonate is difficult to be dissolved in water, but in acidic solutions of S1 or S2, it can be dissolved thoroughly. So we give the concentration of Calcium as above. In diluted solutions these cations were diluted correspondingly, such that their pH did not change.

6. Test solution 1 (S1, pH 3.9), prepared using citric acid (18.0 g), citrate magnesium (1.0 g), calcium carbonate (0.5 g), magnesium carbonate (7.5 g), D-gluconic-acid (3.0 g), dissolved in 100 ml. distilled water, and processed as described for S2. The concentrations of these ingredients are shown in Table 4).

We also altered the pH of these solutions to 4, 5, 7, and 8.5 to test their effectiveness and added different cations (Na^+^, by adding 302.7 mmol/L citrate sodium 10 ml. into 100 ml. of the initial solution; K^+^, by adding 302.7 mmol/L citrate potassium 10 ml. into 100 ml. of the initial solution; Ca^2+^, by adding 454.5 mmol/L calcium chloride anhydrous 10 ml. into 100 ml. of the initial solution). S1 and S2 were formulated according to different dilutions (1:1, 1:2, 1:3, 1:4). The pH of these changed solutions had no detectable variation. After stones were immersed in these solvents for 24, 48 and 72 h, reduction in weight, percentage weight loss, and dissolution rate were determined. Results were analyzed using the Multiple Comparisons Test with the alpha set at 0.05. Stones remaining after the dissolution period were weighed (gm), and final weights were applied to the following formula:



The formula used to determine the dissolution rate was:



#### Chemolysis

Every stone was placed in a vessel containing solvents, which were held at a constant temperature of 37? using a thermostat. Solvents were exchanged 2 times per day at 6 ml/100 mg per stone each time. The pH values were constantly controlled by a pH meter (Mettler Toledo 320-S, sensitivity 0.01). The weight of the calculus was measured continuously by Electronic Semi-micro-, Analytical and Precision Balances (Sartorius BP211D-OCE, sensitivity 0.01 mg) and documented online with a computer system (PC windows 486, Software Wedge for window, version 1.1). The analysis represents six stones for each experiment, repeated three independent times.

## Results

**Figure 1:**
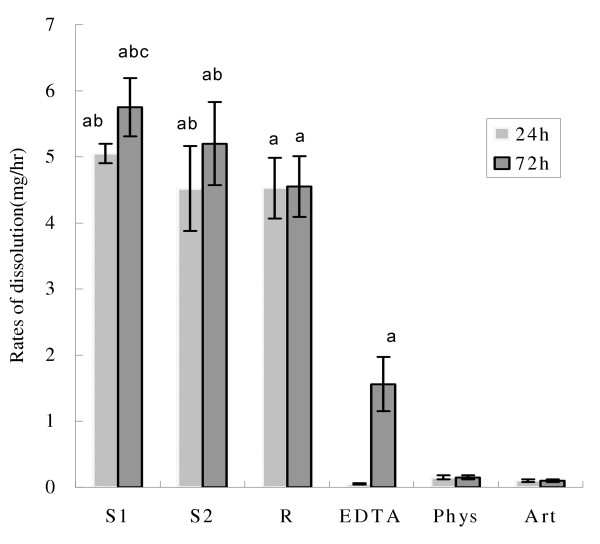
**Dissolution rates of phosphate calculi in vitro at 24 and 72 h using different solutions. **Phys = physiologic sodium chloride solution, pH 7.0. Art = artificial urine, pH 5.7. EDTA = 0.03 M disodium EDTA+TEA, pH 8.5. R = renacidin, pH 4.0. S1 = test solution 1, pH 4.0. S2 = test solution 2, pH 3.9. ^a^*P *< 0.01 vs Phys and Art. ^b^*P *> 0.05 vs EDTA, Phys and Art. ^c^*P *< 0.05 vs R· ± *s*·n = 18 for each group. For statistics, see results section.

Urological calcium phosphate calculi were poorly dissolved by Phys and Art, and they had a low dissolution rate in EDTA at pH 8.5. The most effective solutions were R, S1 and S2 with 24 h mean dissolution rates: 5.05 ± 0.15 mg/hr (S1), 4.52 ± 0.64 mg/hr (S2), 4.53 ± 0.46 mg/hr (R); 72 h mean dissolution rates: 5.75 ± 0.44 mg/hr (S1), 5.2 ± 0.63 mg/hr (S2) and 4.55 ± 0.46 mg/hr (R) ( ± *s*, *P *< 0.01, R, S1 and S2 vs Phys, Art and EDTA; *P *< 0.05, S1 vs R, LSD-test). The mean percentage weight loss at 72 h was: 40.5 ± 3.67 % (R), 52.1 ± 15.75 % (S1) and 44.4 ± 7.37 % (S2) ( ± *s*, *p *< 0.01 R, S1 and S2 vs Phys, Art and EDTA, LSD-test). Figure 1 shows the average dissolution rates of the 6 different solutions at the end of 72 h.

Table 5 Shows mean weight loss and mean percentage weight loss of stones dissolved by the 6 solutions at the end of 24 and 72 h. ( ± *s*, ^a^*P *< 0.01 Phys, Art and EDTA vs R, S1 and S2; ^b^*P *< 0.05 EDTA vs S1, S2 and R.^c^*P *< 0.05 R vs S1).

**Table 5:** Mean weight and percentage weight decrease after 24 and 72 h between groups dissolved with 6 different solutions. Phys = physiologic sodium chloride solution, pH 7.0. Art = artificial urine, pH 5.7. EDTA = 0.03 M disodium EDTA+TEA, pH 8.5. R = renacidin, pH 4.0. S1 = citrate complex 1, pH 4.0. S2 = citrate complex 2, pH 3.9.  ± *s*, ^a^*P *< 0.01 vs R, S1 and S2.^b^*P *< 0.05 vs S1, S2.^c^*P *< 0.05 vs S1. n = 18 for each group. For statistics, see results section.

Groups	Weight loss (mg)		Percentage weight loss (%)	
		
	24 h	72 h	24 h	72 h
Phys	3.52 ± 0.73^a^	18.15 ± 13.15^a^	0.00 ± 0.00^a^	1.33 ± 1.53^a^
Art	2.48 ± 0.37^a^	7.44 ± 1.9^a^	0.00 ± 0.00^a^	0.00 ± 0.00^a^
EDTA	43.78 ± 6.23^ab^	153.6 ± 20.84^ab^	4.67 ± 2.08^ab^	19.00 ± 5.19^ab^
R	114.4 ± 8.92^c^	346.14 ± 26.56^c^	13 ± 1.35^c^	40.5 ± 3.67^c^
S2	127 ± 23.09	408.11 ± 62.94	13.5 ± 2.76	44.4 ± 7.37
S1	138.5 ± 31.09	439.5 ± 102.00	5.77 ± 1.29	52.1 ± 15.75

**Figure 2:**
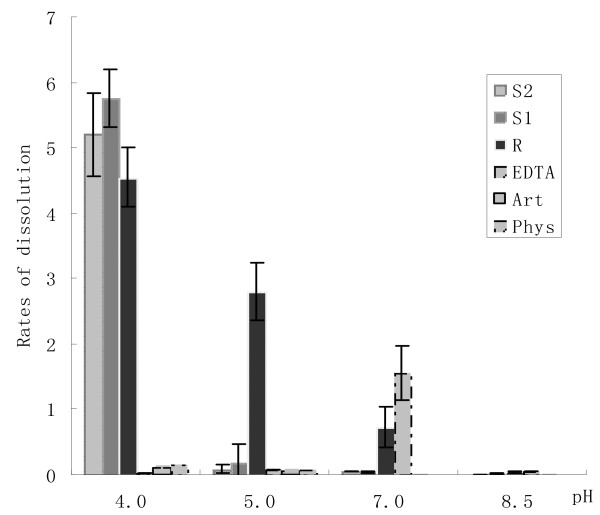
**Effectiveness of six solvents at different pH (mg/hr). **Phys = physiologic sodium chloride solution, Art = artificial urine, EDTA = 0.03 M disodium EDTA+TEA, R = renacidin, S1 = test solution 1, S2 = test solution 2.

The dissolution rates were pH dependent. Even Phys and Art were effective to some extent at pH 4. The addition of 1 mol/L sodium hydroxide 10 ml into S1 or S2 100 ml, though not enough to lead to a detectable pH rise, would make the solutions cloudy and greatly reduce their effectiveness. At pH 5.0 they were nearly ineffective, as also observed at pH 7.0, 8.5. However, the dissolution rate of EDTA increased at an elevated pH value. At pH 8.5, EDTA approached a maximum dissolution rate with 72 h mean 1.56 ± 0.05 mg/hr (Figure 2).

The addition of Na^+^, K^+ ^or Ca^2+ ^(302.7 mmol/L Sodium citrate 10 ml, 302.7 mmol /L Potassium citrate 10 ml or 454.5 mmol/L Calcium chloride dihydrate 10 ml into initial solution 100 ml, respectively) caused no significant change in dissolution rate.

**Figure 3:**
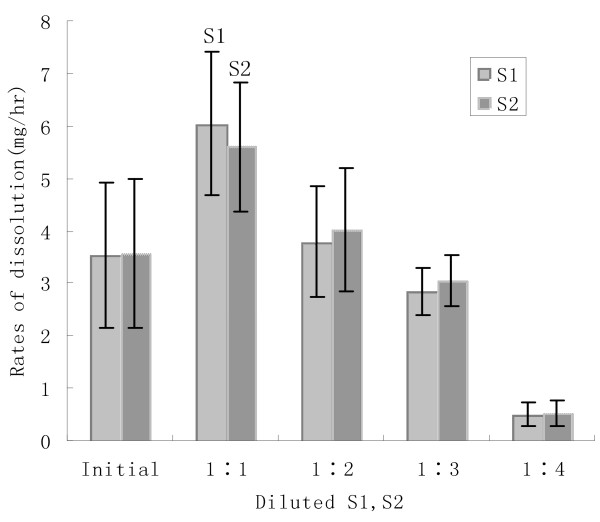
Concentration and effective of S1 and S2

Diluted solutions demonstrated an interesting result. Diluted twice, S1 and S2 were more effective than their initial solutions with 72 h mean dissolution rates: 6.04 ± 1.36 mg/hr (S1), 5.60 ± 1.23 mg/hr (S2) ( ± *s*, *P *< 0.05, S1 and S2 vs R). The effectiveness of three times diluted S1 or S2 was the same as the initial solution. When diluted further, the effectiveness was reduced gradually until nearly 0 after solutions were diluted five times (Figure 3).

## Discussion

Chemolysis is useful for eliminating cystine stones as well as for cases in which lithotripsy or endourology is considered to be difficult or risky. It proved to be a useful method for reducing staghorn stones before performing lithotripsy [6]. The type of chemolytic solution is dependent upon the composition of the stone and must be regarded as an effective adjuvant treatment [7]. Calcium phosphate can be dissolved with Suby or R, but the treatment is often tedious and time consuming. Calcium oxalate, the major urinary stone component, cannot be dissolved by these solutions. EDTA and other strong calcium chelators cannot be used because of their local toxicity. Certain enzymes can digest the organic matrix of the stone [8]. The first attempts to dissolve calcium stones were done by Hellstrom and Albright in 1930. They used citric acid for phosphate calculi, but this proved irritating to tissues. Suby and Albright modified the solution by adding magnesium oxide and sodium carbonate (Suby's G solution) [9,10] to reduce injury to the rabbit bladder mucosa even though adding this cation reduced the speed of dissolution of struvite Mg (NH_4_) PO_4_. A contradictory conclusion was been made by a Dutch study [11] that magnesium in R promotes stone dissolution by cation exchange with calcium in apatite [12].

Our S1 and S2 solutions were similar to R although actual formulas were different. First, we added D-gluconic acid-lactone or D-gluconic-acid as new chelators which bind with calcium well by their special trait. Second, we used new ingredients and ingredients at different weights. The higher density of our solutions than R may be one of the factors which enhanced stone dissolution rates, but it may prevented further dissolution. When diluted twice, the solutions may have gained more space to accommodate cations than the initial solutions and achieve better effectiveness (*P *< 0.05, S1 and S2 vs R). The further diluted solutions had a lower concentration suggesting that less ingredient took part in the reaction thus accounting for a lower dissolution rate. So, we think twice diluted S1 and S2 may be more useful than their initial solutions.

The dissolution of the majority of the stones in this study can be attributed on the basis of their reaction equation:

3Ca^+2 ^+ 2 Citrate→CaCitrate→Ca^2+ ^+ CaCitrate

The citrate in the solution binds with the calcium component of the stone producing calcium citrate, thus preventing crystallization. The dissolution process may be brought about by the combination of the above reaction.

As the solubility of calcium phosphate is very pH dependent, the acidification of the urine by the incorporation of citric acid produce a pH between 3.0–4.0, the ideal pH for dissolution as described by Albright et al [9,10]. When the pH is elevated, the citrate will react with cations thus losing its effectiveness. On the other hand, excessive alkalization may lead to the formation of calcium phosphate calculi. Added minor quantities of cations (Na^+^, K^+ ^or Ca^2+^) in S1 and S2 apparently causes no significant change to their dissolution rate.

## Conclusion

The study has shown that solutions S1 and S2 can dissolve calcium phosphate stones effectively in vitro at a precise dilution of their chemical components. Based on these findings, it is suggested that S1 and S2 may become useful complements to modern techniques of stone fragmentation such as extracorporeal shock wave lithotripsy and percutaneous surgery. Their roles may be suited for the treatment of infectious stones with a CaOx content of less than 10%. However, their safety profiles should be further investigated in order to support their use in subsequent human trials.

## Competing interests

The author(s) declare that they have no competing interests.

## Authors' contributions

Xiang-bo Zhang participated in the design of the study, in the sequence alignment, carried out the experiment, performed the statistical analysis and drafted the manuscript. Zhi-ping Wang conceived of the study, and participated in its design and coordination and helped to draft the manuscript. All authors read and approved the final manuscript. Jian-min Duan, Jian-zhong Lu, Bao-liang Ma, participated in the sequence alignment.

## Pre-publication history

The pre-publication history for this paper can be accessed here:


